# Management of Cystoid Macular Edema in Retinitis Pigmentosa: A Systematic Review and Meta-Analysis

**DOI:** 10.3389/fmed.2022.895208

**Published:** 2022-05-16

**Authors:** Chen Chen, Xia Liu, Xiaoyan Peng

**Affiliations:** ^1^Department of Ophthalmology, The Second People's Hospital of Yunnan Province (Affiliated Hospital of Yunnan University, Fourth Affiliated Hospital of Kunming Medical University), Kunming, China; ^2^Beijing Ophthalmology and Visual Science Key Laboratory, Beijing Institute of Ophthalmology, Beijing, China; ^3^Beijing Tongren Eye Center, Beijing Tongren Hospital, Capital Medical University, Beijing, China; ^4^Yunnan Clinical Medicine Center for Ocular Disease, Yunnan Eye Institute, Kunming, China; ^5^Key Laboratory of Yunnan Province for the Prevention and Treatment of Ophthalmic Diseases, Yunnan Eye Institute, Kunming, China

**Keywords:** retinitis pigmentosa, cystoid macular edema, carbonic anhydrase inhibitors, steroids, systematic review, meta-analysis

## Abstract

**Background:**

To date, various treatments for cystoid macular edema (CME) in retinitis pigmentosa (RP) have been reported. We performed a systematic review and meta-analysis to evaluate the efficacy and safety of current treatments for RP-CME.

**Methods:**

PubMed, Embase and the Cochrane library were searched from inception to August 2021. ClinicalTrials.gov, WHO ICTRP and ISRCTN were also searched for relevant studies. Only studies published in English were included. The RoB 2 tool was used to evaluate the risk of bias of randomized controlled trials (RCTs), and the MINORS scale was used to assess the methodological quality of non-RCTs. Review manager (Revman) was used to pool the data. The primary outcomes included the change of central macular thickness (CMT) and best-corrected visual acuity (BCVA) from baseline. The secondary outcomes included fluorescein angiography (FA) leakage, rebound of CME and adverse effects.

**Results:**

Thirty-two studies were included in the current systematic review and 7 studies were used for meta-analysis. Treatments for RP-CME included oral and topical carbonic anhydrase inhibitors (CAIs), systematic and local steroids, anti-VEGF therapy, NSAIDS, grid LASER photocoagulation, subliminal micropulse LASER, vitrectomy, lutein supplement and oral minocycline. CAIs and local steroids were proved to be effective in reducing CMT. The effects of anti-VEGF reagents varied among studies. Regarding other treatments, only one study for each method fitted the inclusion criteria, so the evidence was very limited.

**Conclusion:**

Topical CAIs, oral CAIs and local steroids are effective in treating RP-CME. However, due to the overall inferior design and small patient number of the included studies, the quality of evidence was poor. Systematic steroids, LASER, NSAIDS and vitrectomy may also be effective, nevertheless, considering the limited number of studies, no conclusion could be drawn regarding these treatments. More well-designed and conducted studies are needed in this field.

**Systematic Review Registration:**

https://www.crd.york.ac.uk/prospero/display_record.php?ID=CRD42021273979, identifier CRD42021273979.

## Introduction

Retinitis pigmentosa (RP) is an inherited retinal dystrophy that primarily involves the rod photoreceptors, leading to low vision and blindness. The incidence of RP is 1 in 4,000 (1). To date, over 100 RP-causing genes with diverse mutations have been identified (2). At the early stage, RP is characterized by the constriction of visual field, while the central vision might be reserved. Cystoid macular edema (CME) is observed in 10–50% of RP patients when searched with optical coherence tomography (OCT) (3, 4), with the pathological mechanisms include the blood-retina barrier (BRB) breakdown, retinal pigment epithelium (RPE) pumping dysfunction, inflammatory responses and vitreous traction (4). When macular edema occurs in RP patients, the central vision will be impaired.

In 1988, Cox et al. reported the application of acetazolamide (AZM) in a group of patients with CME due to different diseases. Among the 6 included RP-CME patients, 4 responded to the drug, as indicated by improved visual acuity (VA) and reduced fluorescein angiography (FA) leakage in the macular region (5). Later, Fishman et al. reported the efficacy of oral methazolamide and topical dorzolamide in the treatment of RP-CME (6, 7). Nowadays, carbonic anhydrase inhibitors (CAIs) including AZM, methazolamide and dorzolamide are recommended as the first-line choice of drugs for RP-CME (4, 8). On the other hand, steroids were also believed to be useful. Oral and local steroids were reported to be effective in reducing central macular thickness (CMT) as well as improving visual acuity (VA) in RP-CME patients (9, 10). In recent years, the application of slow-releasing intravitreal steroids has proved beneficial, with minimal systematic side effects (11). Other treatments for RP-CME including anti-VEGF therapy, LASER treatment and vitrectomy have also been reported, with varied results in clinical trials.

Two systematic reviews and one meta-analysis have been published regarding the treatment of RP-CME, with results of studies up to 2016 summarized and analyzed (4, 8, 12). Nevertheless, during the past 5 years, more evidence has been published on the application of CAIs (13–18), steroids (10, 11, 13, 14, 16, 19), anti-VEGF therapy (20), and LASER treatment (21). Therefore, we conducted this updated systematic review and meta-analysis, to summarize the existing evidence on the treatment of RP-CME.

## Materials and Methods

### Protocol and Registration

The current systematic review and meta-analysis was conducted according to the PRISMA guideline (Supplementary Data Sheet 1) (22). This work was registered in PROSPERO (registration number CRD42021273979).

### Search Strategy

We searched PubMed, Embase and the Cochrane library from inception to August 2021. The websites of ClinicalTrials.gov, WHO ICTRP and ISRCTN were also searched. Combinations of various forms of the keywords “retinitis pigmentosa” and “macular edema” were used in the search process, and the detailed search strategy was in Supplementary Data Sheet 2. Duplicates were identified and removed by the Endnote software (Clarivate Analytics, USA), followed by removal of irrelevant records by manual screening of the titles and abstracts. For remaining records, the full texts were retrieved and assessed against the inclusion criteria. Two reviewers (Chen and Liu) searched the databases and screened the records independently. Disagreements were solved by consulting the third reviewer (Peng).

### Inclusion and Exclusion Criteria

The inclusion criteria (PICOS) were: (1) Participants (P): RP patients with CME. (2) Interventions (I): any intervention that aimed to treat CME. (3) Comparison (C): both comparative studies and single-arm studies were included. (4) Outcomes (O): primary outcomes included the change of CMT and best-corrected visual acuity (BCVA) from baseline. Secondary outcomes included FA leakage, rebound of CME and adverse effects. (5) Type of study (S): any study, prospective or retrospective, that approached the management of RP-CME were included.

The exclusion criteria were: (1) Studies that had <5 patients; (2) Studies published in languages other than English.

### Data Extraction and Quality Assessment

The following data were extracted from each included study: first author, publication year, location where the study was conducted, study type, participants, age, interventions, number of patients/eyes, follow-up duration and outcome measurements. For randomized controlled trials (RCTs), the updated Cochrane risk-of-bias tool (RoB 2) was used to assess the methodological quality (23). For non-randomized comparative studies and single-arm studies, the methodological index for non-randomized studies (MINORS) was used to evaluate the study quality (24). The ideal MINORS score was 16 for single-arm studies and 24 for comparative studies. Two reviewers (Chen and Liu) performed data extraction and quality assessment independently. Consensus was reached by consulting the third reviewer (Peng).

### Statistical Analysis

Because of substantial heterogeneity among the included studies, we only pooled the data from several single-arm trials exploring the efficacy of CAIs treatment. CMT values at the last visit were used for analysis. Data from prospective and retrospective studies were pooled separately. For steroids treatment, the data from different studies were put together in a diagram for clarity, but were not pooled due to heterogeneity. For other treatments including anti-VEGF therapy, LASER treatment, vitrectomy, lutein supplement and NSAIDS eyedrops, a systematic review was performed instead of meta-analysis. Review manager (the Cochrane Collaboration, UK) was used to pool the data and generate the figures. Mean Difference (MD) (for CMT change) or Risk Difference (RD) (for responder proportion) was calculated. Subgroup analysis was carried out regarding different means of CAIs treatment (oral or topical). The heterogeneity among included trials was assessed with I^2^ statistics. If the heterogeneity was low (I^2^ < 50%), fixed effect model was employed to pool the data. If the heterogeneity was substantial (I^2^ > 50%), random effect model was used. A *p* < 0.05 was considered statistically significant for treatment effects.

## Results

### Study Characteristics

**Figure 1:**
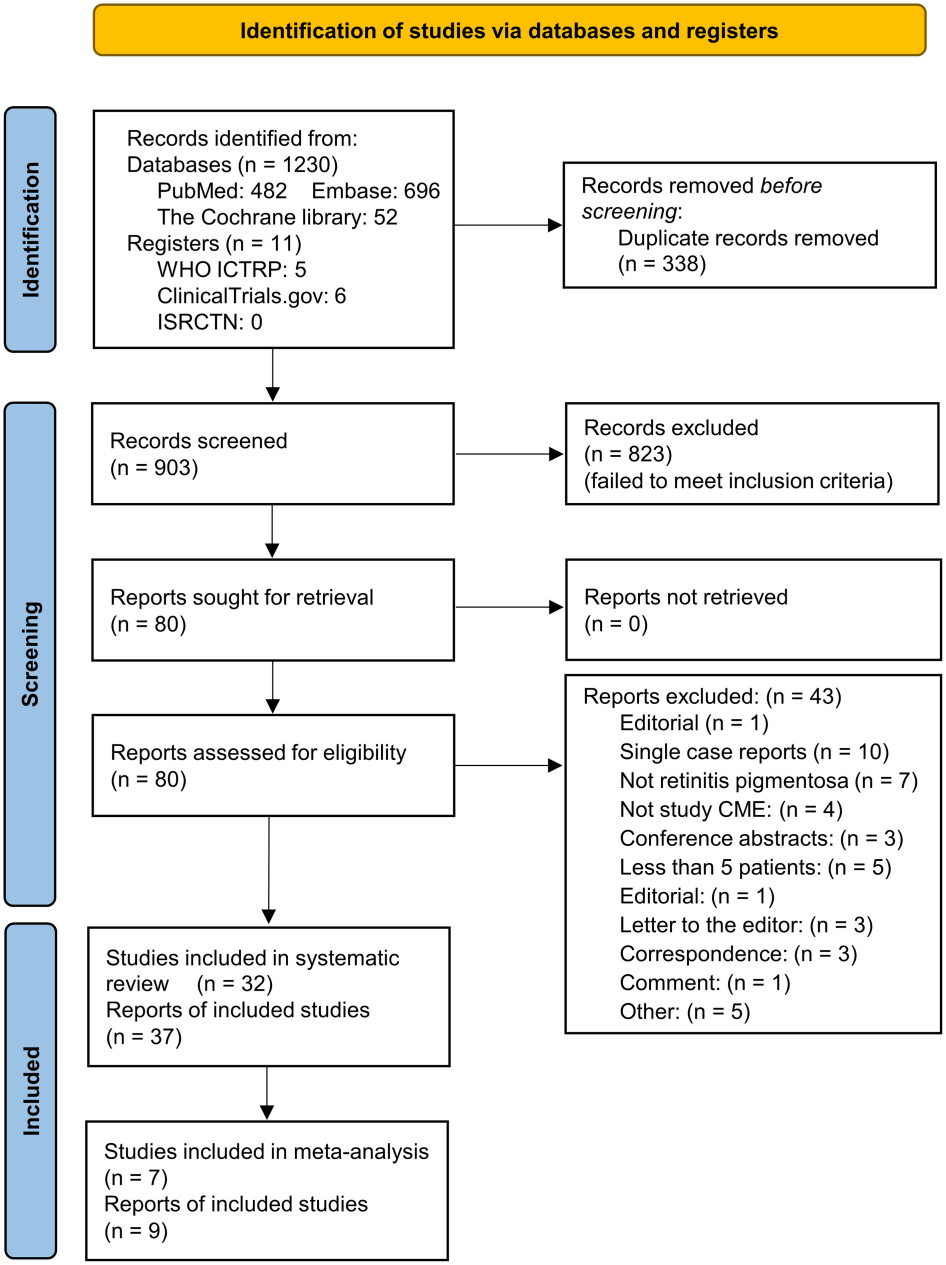
Flow diagram of literature search and records screening.

**Figure 2:**
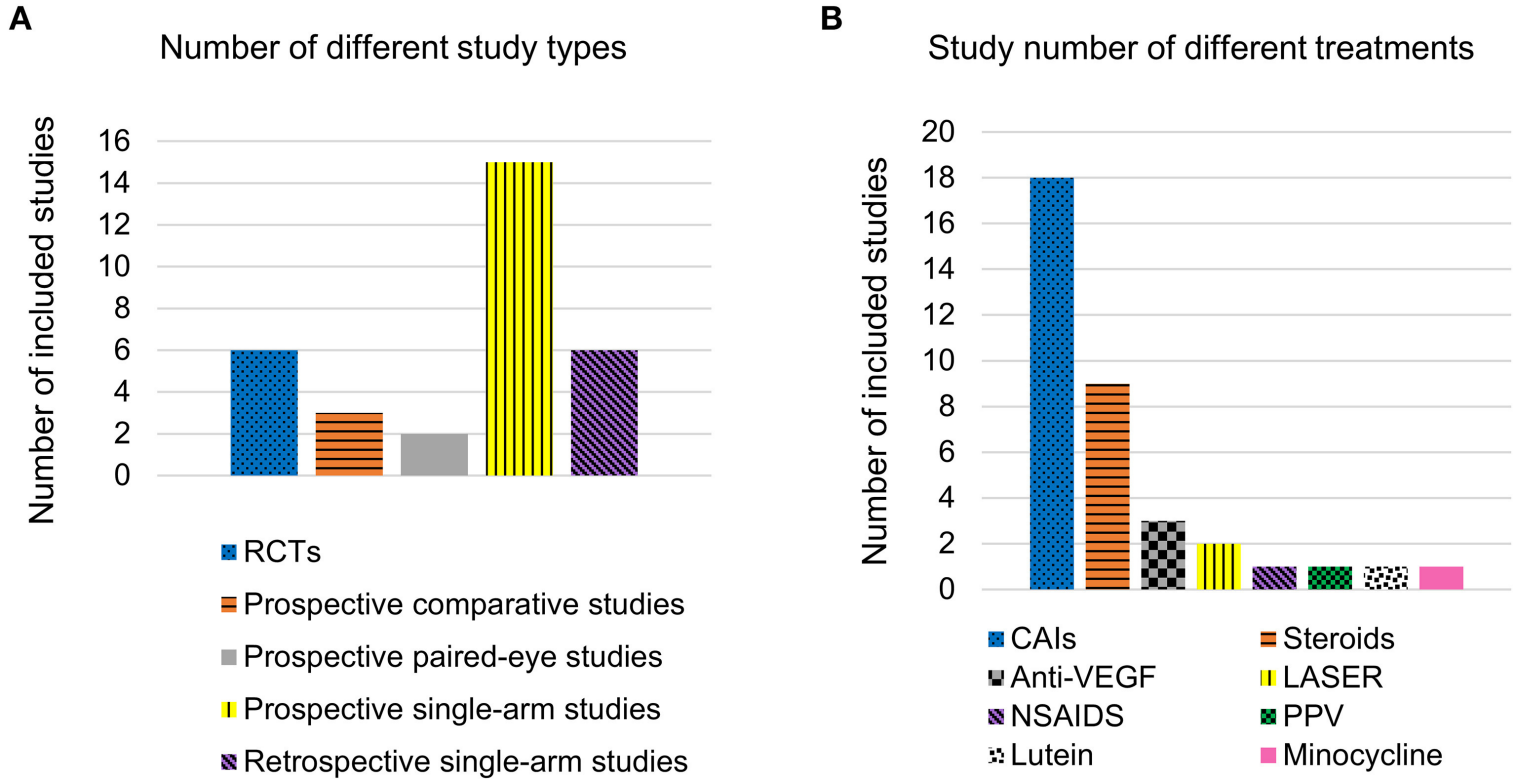
Distribution characteristics of included studies. **(A)** Number of different study types; **(B)** Study number of different treatments.

During databases and registers searching, 1,241 records were identified. After removal of 338 duplicates, 903 titles and abstracts were screened. Eighty full-text records were evaluated according to the inclusion criteria, and 43 records were excluded with reasons (see Figure 1). A total of 32 studies (37 reports) were included in the qualitative synthesis, and 7 studies (9 reports) were used for meta-analysis (Figure 1). Among the included studies, 6 were RCTs (5 crossover and 1 parallel design), 3 were prospective comparative studies, 2 were prospective paired-eye studies, 15 were prospective single-arm studies, and 6 were retrospective single-arm studies (Figure 2A). Treatments for RP-CME included CAIs (14 studies), steroids (6 studies), CAIs compared with steroids (2 studies), steroids (betamethasone) additional to CAIs (1 study), CAIs compared with NSAIDS (ketorolac) (1 study), anti-VEGF therapy (3 studies), LASER treatment (2 studies), pars plana vitrectomy (PPV) (1 study), lutein supplement (1 study) and oral minocycline (1 study) (Figure 2B). Detailed study characteristics were presented in Table 1.

**Table 1:** Characteristics of included studies.

**Treatment**	**First author /year**	**Location**	**Study design**	**Participants**	**Age (year) (mean, range)**	**Intervention**	**Patients/Eyes**	**Follow-up duration**	**Outcome measurements**
CAIs	Cox, 1988	UK	Prospective single arm study	CME due to various reasons (6 RP-CME)	28–84 for responders	Oral acetazolamide 500 mg/d, then cyclopenthiazide 0.5 mg/d	Total 41 patients (RP-CME: 6/12)	16 w	BCVA, FA grade of CME
	Fishman, 1989	US	RCT (crossover design)	RP-CME	45 (29–79)	Oral acetazolamide 500 mg/d vs. placebo	12/24	4 w-21 w	BCVA, FA grade of CME, subjective improvement
	Orzalesi, 1993	Italy	Prospective single arm study	RP (7 RP patients, 5 have CME)	23–60	Oral acetazolamide 500 mg/d, tapered to 125 mg every 3 days	5/9	3 w-16 m	BCVA, FA grade of CME, macular threshold
	Fishman, 1994 Fishman 1993	US	RCT (crossover design, multicenter)	RP-CME	NR (inclusion criteria 18–65)	Oral methazolamide 50 mg bid vs. placebo	17/34	10 w-5 m	BCVA, FA grade of CME, subjective improvement
	Grover, 1997	US	RCT (crossover design)	RP-CME	44 (37–53)	Topical 2% dorzolamide vs. placebo, then oral acetazolamide 500 mg/d	5/10	26 w	BCVA, FA grade of CME
	Moldow, 1998	Denmark	RCT (crossover design)	CME due to RP and US (9 patients, 7 have CME)	38.7 (24–63)	Oral acetazolamide 250 mg bid vs. placebo	7/14	4 w	BCVA, FA grade of CME, penetration ratio of fluorescein, AEs
	Chung, 2006	Korea	Prospective single arm study	RP-CME	28–66	Oral acetazolamide 125 mg or 250 mg daily for 4–12 m	10/20	4–12 m	CFT, BCVA, FA leakage
	Apushkin, 2007	US	Prospective single arm study	RP-CME	21–48	Oral acetazolamide 500 mg/d for 8–12 w	6/12	8–22 w	BCVA, FT, FZT
	Grover, 2006 Fishman 2007	US	Prospective single arm study	RP-CME	38 (16–62)	topical 2% dorzolamide tid	15/28	1–15 m	BCVA, FT, FZT
	Genead 2010	US	Retrospective single arm cohort study	CME due to RP and US	38.2 (19–67)	Topical 2% dorzolamide tid	32/64	6–58 m	BCVA, CFZ thickness, responder proportion
	Ikeda, 2012 Ikeda 2013	Japan	Prospective single arm study	RP-CME	43 (20–60)	topical 1% dorzolamide tid	10/18	12–18 m	BCVA, CST, MD and macular sensitivity
	Liew, 2015	UK	Retrospective single arm cohort study	RP-CME	Oral: 36.0 topical: 45.4	oral acetazolamide 250 mg bid or 500 mg qd, or topical 2% dorzolamide tid	Oral: 17/32 topical: 64/115	1.5–12 m	BCVA, CSF thickness, responder proportion
	Reis, 2015	Portugal	RCT	CME due to RP and US	Dorzolamide: 43.54 ketorolac: 41.80	2% dorzolamide 3 drops daily vs. 0.5% ketorolac 4 drops daily	18/28 (dorzolamide: 9/13 ketorolac: 9/15)	12 m	BCVA, FT, FZT
CAIs	Strong, 2019	UK	Retrospective single arm cohort study	RP-CME	48 (17–79)	Oral acetazolamide 250 mg bid or topical dorzolamide/brinzolamide tid	25/43 (acetazolamide: 4 eyes, dorzolamide/ brinzolamide: 39 eyes)	3–9 m	CMT, BCVA change, responder proportion, CME fluid distribution
	Shimokawa, 2020; Shimokawa, 2021	Japan	Retrospective single arm cohort study	RP-CME	53	1.0% dorzolamide eyedrop tid	47/66	0.8–10.1 y	Responder proportion, CME fluid distribution, macular sensitivity
	Veritti, 2020	Italy	Prospective, non-randomized, propensity-score-matched, comparative study	RP-CME, with CRT>350 μm	Dexamethasone implant: 38.3 oral acetazolamide: 36.7	Oral acetazolamide 500 mg/day vs. dexamethasone implant (0.7 mg, Ozurdex)	60/60 (oral acetazolamide: 30/30, dexamethasone implant: 30/30)	12 m	CRT, BCVA, number of injections, AEs
	Park, 2020; Park, 2021	Korea	Randomized, non-controlled, paired-eye, single crossover study	RP with bilateral CME, CMT>250 μm	51.5 (34–66)	Topical 2% dorzolamide vs. intravitreal dexamethasone implant (0.7 mg, Ozurdex)	14/28	12 m	CMT, BCVA, IOP
Steroids	Giusti, 2002	Italy	Prospective single arm study	RP-CME	42.7	Oral deflazacort 30 mg/d, tapered for a total 12 m	10 patients	12 m	BCVA (far and near), FA grade of CME, MD and retinal sensitivity
	Ozdemir, 2005	Turkey	Prospective single arm study	RP-CME unresponsive to oral acetazolamide	33.2 (25–41)	Intravitreal injection of 4 mg (0.1 ml) triamcinolone acetonide	5/5	6–8 m	VA, CMT
	Scorolli, 2007	Italy	Prospective, non-randomized, controlled study	RP-CME	treatment: 40.2 (28–54) control: 39.5	Intravitreal injection of 4 mg (0.1 ml) triamcinolone acetonide vs. observation	Treatment: 20/20 control:20/20	12 m	CMT, BCVA, IOP
	Sudhalkar, 2017	India	Prospective single arm study	RP-CME with incomplete or no response to CAIs	43–56	Intravitreal dexamethasone implant (0.7 mg, Ozurdex)	5/6	2 y	CDVA, CST, IOP, number of injections
	Mansour, 2018	Lebanon	Retrospective single arm multicenter study	RP-CME (previously untreated or treated)	32.7 (16–57)	Intravitreal dexamethasone implant (0.7 mg, Ozurdex)	34/45	1–48 m	CMT, BCVA, IOP
	Kitahata, 2018	Japan	Retrospective single arm cohort study	Persistant RP-CME	39.4 (16–51)	Topical 0.1% betamethasone tid or qid (in addition to previous topical dorzolamide or brinzolamide/bromfenac)	10/16	3–58 m	BCVA, CFT
	Karasu, 2020	Turkey	Prospective single arm study	RP-CME unresponsive to CAIs	36.25 (13–63)	Subtenon triamcinolone acetonide (1 ml: 40 mg)	42/48	4–6 m	CMT, BCVA, IOP
Steroids	Veritti, 2020	Italy	Prospective, non-randomized, propensity-score-matched, comparative study	RP-CME CRT>350 μm	Dexamethasone implant: 38.3 oral acetazolamide: 36.7	dexamethasone implant (0.7 mg, Ozurdex) vs. oral acetazolamide 500 mg/day	60/60 (oral acetazolamide: 30/30, dexamethasone implant: 30/30)	12 m	CRT, BCVA, number of injections, AEs
	Park, 2020; Park, 2021	Korea	Prospective, paired-eye, crossover study	RP with bilateral CME, CMT>250μm	51.5 (34–66)	Intravitreal dexamethasone implant (0.7 mg, Ozurdex) vs. 2% topical dorzolamide	14/28	12 m	CMT, BCVA, IOP
Anti-VEGF	Artunay, 2009	Turkey	Prospective, non-randomized, controlled study	RP with persistent CME despite previous medication	Treatment: 36.6 (29–52) control: 39.6 (26–55)	intravitreal ranibizumab 0.5 mg (single injection) vs. observation	Treatment: 15/15 control:15/15	6 m	BCVA, CFT
	Yuzbasioglu, 2009	Turkey	Prospective single arm study	RP with persistent CME despite previous medication	44.14 (25–69)	Intravitreal bevacizumab (1.25 mg/0.05 ml)	7/13	6–14 m	CMT, VA, number of injections
	Strong 2020	UK	Prospective single arm study	RP-CME	43.3	Intravitreal aflibercept (50 μl, 2 mg) (3+TAE)	30/30	12 m	CMT, BCVA, retinal sensitivity, AEs
LASER	Newsome, 1987	US	Prospective paired-eye study	RP-CME	34.2 (19–60)	Grid laser photocoagulation	16/16	4–21 m	BCVA, FA leakage
	Arslan, 2021	Turkey	Prospective single arm study	RP-CME unresponsive to CAIs, CMT>500 μm	38.8 (18–67)	Subliminal micropulse yellow laser	29/32	12 m	CMT, BCVA, subjective improvements
Vitrectomy	Garci'a-Arumi', 2003	Spain	Prospective single arm study	RP-CME unresponsive to oral acetazolamide	26–48	Pars plana vitrectomy + inner limiting membrane removal + gas tamponade	8/12	12 m	BCVA, foveal thickness, FA leakage
NSAIDS	Reis, 2015	Portugal	RCT	CME due to RP and US	Ketorolac: 41.80 dorzolamide: 43.54	0.5% ketorolac 4 drops daily vs. 2% dorzolamide 3 drops daily	18/28 (ketorolac: 9/15 dorzolamide: 9/13)	12 m	BCVA, FT, FZT
Lutein	Adackapara, 2008	US	RCT (crossover design)	RP	51 (23–67)	Oral lutein 10 or 30 mg/d vs. placebo	Total 39/77 RP-CME 19/36	48 w	BCVA, central thickness
Minocycline	NCT02140164 PI: Dr Cukras, completed 2016	US	Prospective single arm study (phase I/II clinical trial)	RP-CME	27.7	Oral minocycline 100 mg bid for 12 m	7 participants, 5 completed	12 m	Change of CMT, microperimetry, visual field and VA. AEs

*CAIs, carbonic anhydrase inhibitors; RCT, randomized controlled trial; RP, retinitis pigmentosa; CME, cystoid macular edema; BCVA, best-corrected visual acuity; FA, fluorescein angiography; CMT, central macular thickness; CFT, central foveal thickness; FT, foveal thickness; FZT, foveal zone thickness; CFZ, central foveal zone; MD, mean deviation; CSF, central subfield; IOP, intraocular pressure; CST, central subfield thickness; CDVA, corrected distance visual acuity; CRT, central retinal thickness; AE, adverse events; VA, visual acuity*.

### Quality Assessment

**Figure 3:**
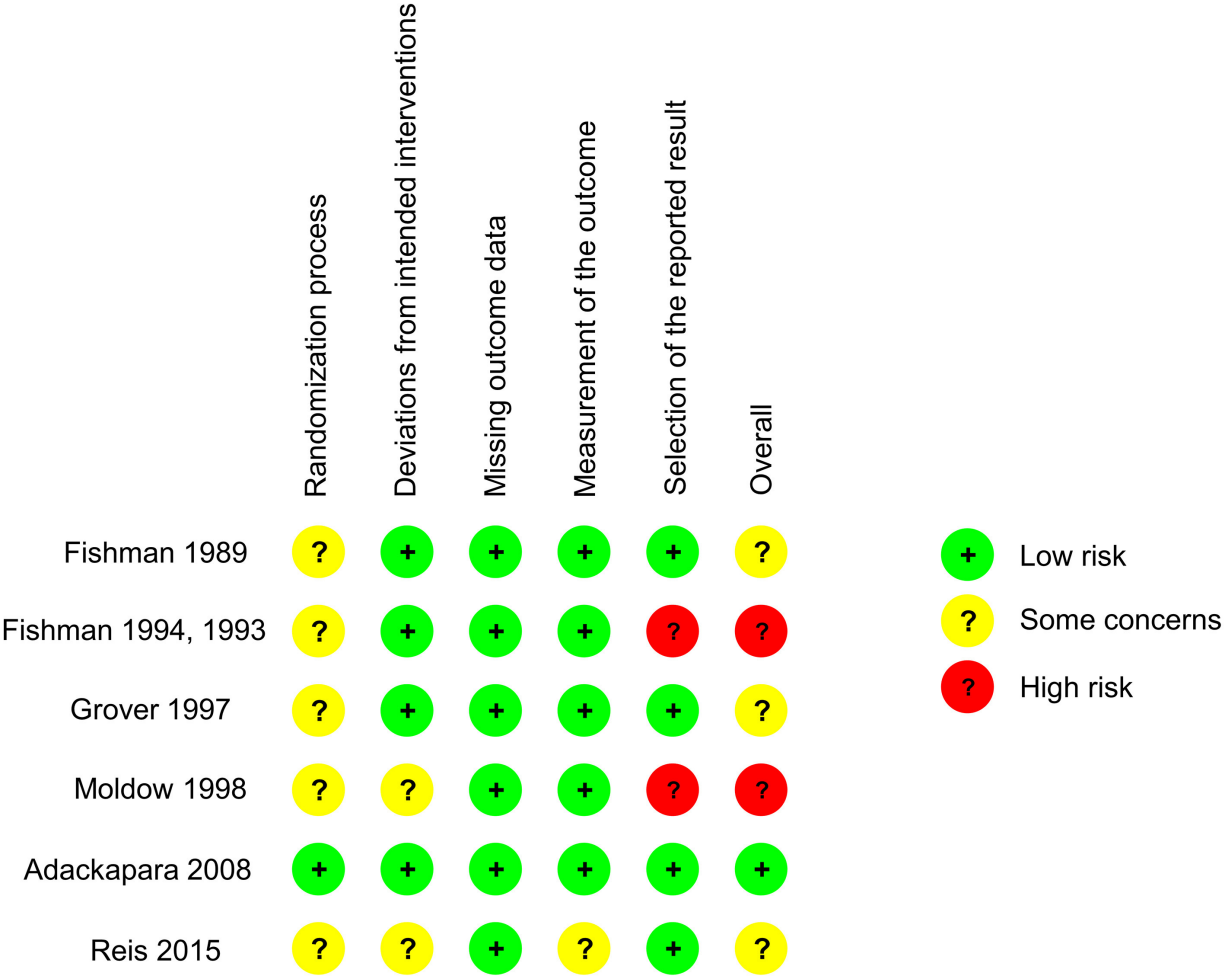
Summary of risk of bias assessment for randomized controlled trials (RCTs) employing RoB 2 tool.

For methodological quality assessment, the 6 RCTs were evaluated with the RoB2 tool, and the remaining 26 studies were assessed using the MINORS scale. Of the 6 RCTs, 1 was determined to be at low risk of bias, 3 were determined to be of some concerns because of potential bias in the randomization process (1 of the 3 also had potential bias in deviations from intended interventions and measurement of the outcome), 2 were determined to be at high risk of bias due to the selection of the reported result (Figure 3). By MINORS scale, the 5 prospective comparative studies (including the 2 paired-eye studies) were scored 15–20 out of an ideal score of 24. The 15 prospective single-arm studies were scored 8–13, and the 6 retrospective single-arm studies were scored 5–8 out of an ideal score of 16 (Table 2).

**Table 2:** Quality assessment of non-RCT studies using the MINORS scale.

**References**	**A clearly stated aim**	**Inclusion of consecutive patients**	**Prospective collection of data**	**Endpoints appropriate to the aim of the study**	**Unbiased assessment of the study endpoint**	**Appropriate follow-up period**	**Loss to follow up less than 5%**	**Prospective calculation of the study size**	**An adequate control group**	**Contemporary groups**	**Baseline equivalence of groups**	**Adequate statistical analyses**	**MINORS Score**
Newsome and Blacharski (25)	2	0	2	2	2	1	2	0	1	2	1	2	17/24
Cox et al. (5)	2	2	2	2	0	1	1	0	0	0	0	0	10/16
Orzalesi et al. (26)	2	0	2	2	0	1	2	0	0	0	0	0	9/16
Giusti et al. (9)	2	0	2	2	0	2	0	0	0	0	0	0	8/16
García-Arumí (40)	2	2	2	2	0	1	2	0	0	0	0	0	11/16
Ozdemir et al. (27)	2	0	2	2	0	1	2	0	0	0	0	0	9/16
Chung et al. (28)	2	2	2	2	0	1	2	0	0	0	0	0	11/16
Apushkin et al. (29)	2	0	2	2	2	1	2	0	0	0	0	0	11/16
Grover et al. (30), Fishman and Apushkin (7)	2	1	2	2	1	1	2	0	0	0	0	0	11/16
Scorolli et al. (31)	2	0	2	2	0	2	0	0	1	2	2	2	15/24
Artunay et al. (32)	2	2	2	2	2	1	1	0	1	2	2	2	19/24
Yuzbasioglu et al. (33)	2	0	2	2	0	1	2	0	0	0	0	0	9/16
Genead and Fishman (34)	2	0	0	2	0	1	2	0	0	0	0	0	7/16
Ikeda et al. (35)	2	2	2	2	0	2	1	0	0	0	0	0	11/16
Liew et al. (36)	2	0	0	2	0	1	1	0	0	0	0	0	6/16
Sudhakar et al. (19)	2	2	2	2	0	2	2	0	0	0	0	0	12/16
Mansour et al. (11)	2	0	0	2	0	1	0	0	0	0	0	0	5/16
Kitahata et al. (37)	2	0	0	2	0	1	1	0	0	0	0	0	6/16
Stong et al. (17)	2	2	0	2	0	0	2	0	0	0	0	0	8/16
Karasu (10)	2	2	2	2	0	1	0	0	0	0	0	0	9/16
Shimokawa et al. (15, 18)	2	0	0	1	0	2	2	0	0	0	0	0	7/16
Strong et al. (20)	2	0	2	2	1	2	2	2	0	0	0	0	13/16
Veritti et al. (14)	2	0	2	2	0	2	2	2	2	2	2	2	20/24
Arslan (21)	2	0	2	2	0	2	2	0	0	0	0	0	10/16
Park (13, 16)	2	0	2	2	1	1	1	0	1	1	2	2	15/24
NCT02140164	2	0	2	2	0	2	1	0	0	0	0	0	9/16

### Primary Outcome: Change in CMT

In our included studies, macular thickness was entitled diversely as central macular thickness (CMT), central foveal thickness (CFT), foveal zone thickness (FZT), central subfield thickness (CST), or central retinal thickness (CRT). Here we use CMT throughout this paper for consistency. In the OCT era, macular edema is assessed by OCT measurements of the CMT. In the pre-OCT era, macular edema was evaluated by FA leakage in the macular region. Of the included studies, 22 reported the CMT change after treatment, 8 reported the change in FA leakage, and 2 reported both outcomes.

**Figure 4:**
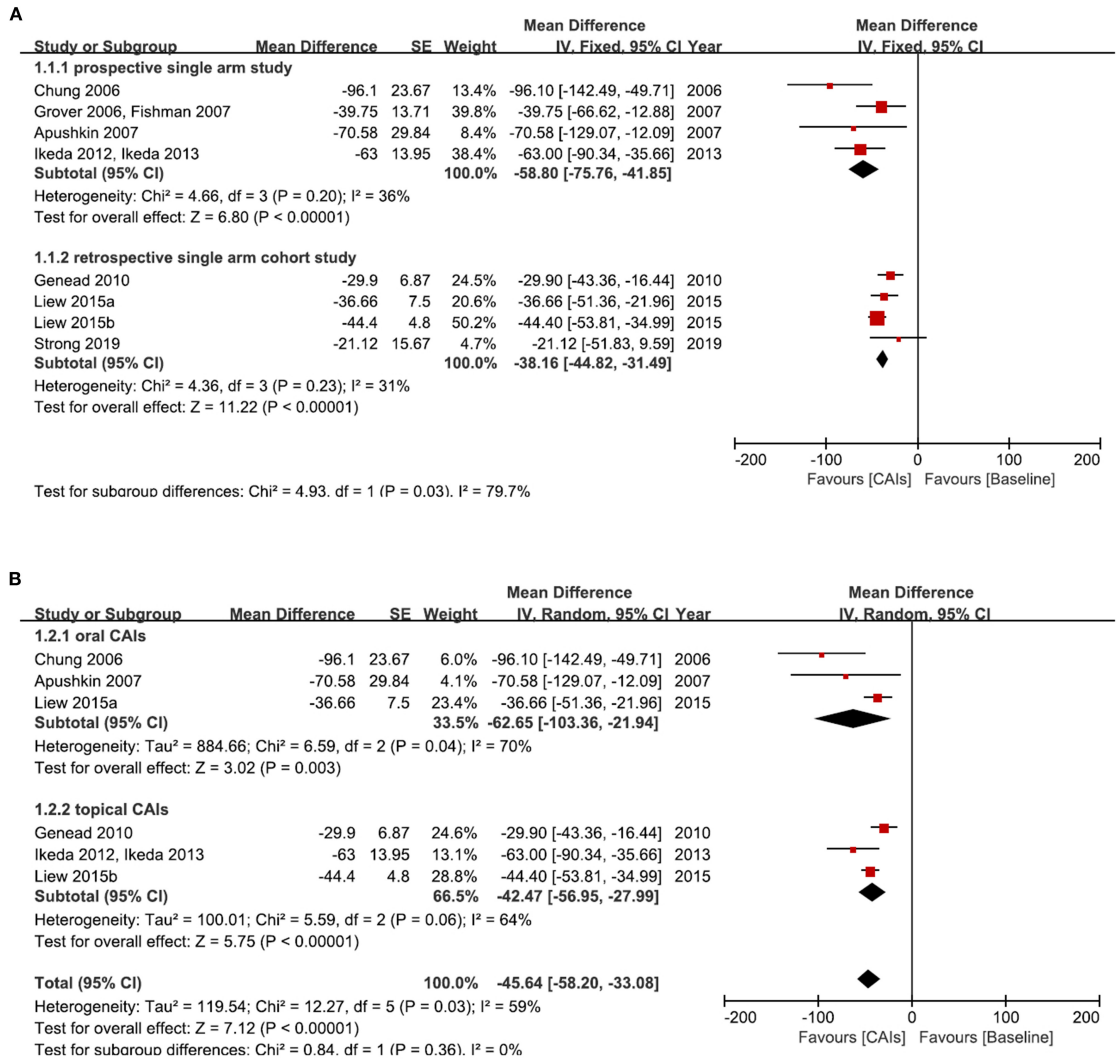
Forest plots for the meta-analysis of change of central macular thickness (CMT) (μm) from baseline after carbonic anhydrase inhibitors (CAIs) treatment. **(A)** Meta-analysis of different study types; **(B)** Subgroup analysis according to different administration methods of CAIs. [For the studies Chung et al. (28), Grover et al. (30)/Fishman and Apushkin (7), Apushkin et al. (29), and Ikeda et al. (35, 39), the change of CMT was calculated from published original individual data; For the study Genead and Fishman (34), the change of CMT was calculated from the mean/standard deviation data before and after treatment; For the study Liew et al. (36), the change of CMT was calculated from the mean/95% CI of CMT reduction in responders and non-responders; For the study Strong 2019, the change of CMT was calculated from the mean/standard deviation data which was extracted from the box plot from the original article by Photoshop software] [*The study Strong 2019 was used for analysis in **(A)** but not in **(B)** because oral and topical CAIs treatment data cannot be separated in this study. The study Grover et al. (30)/Fishman and Apushkin (7) was used for analysis in **(A)** but not in **(B)** because this study may share some same patients with the study Genead and Fishman (34)].

CAIs have been used in clinical trials to treat RP-CME for over 30 years (4, 5, 38). Pooled data from 4 prospective single-arm studies including 41 patients (78 eyes) demonstrated a significant decrease in CMT from baseline after CAIs treatment (CMT values at the last visit were used for analysis) (mean difference: −58.8 μm, 95% CI: −75.76 μm, −41.85 μm, I^2^ = 36%, *P* <0.00001). Data from 3 retrospective cohort studies including 138 patients (254 eyes) also revealed a similar effect of the CAIs (mean difference: −38.16 μm, 95% CI: −44.82 μm, −31.49 μm, I^2^ = 31%, *P* <0.00001) (Figure 4A). Regarding different administration methods, both oral CAIs and topical CAIs significantly decreased CMT (Figure 4B). Generally, CAIs decreased CMT by 45.64 μm from baseline, as demonstrated by the meta-analysis including 5 studies (139 patients and 261 eyes, *P* <0.00001) (Figure 4B).

**Figure 5:**
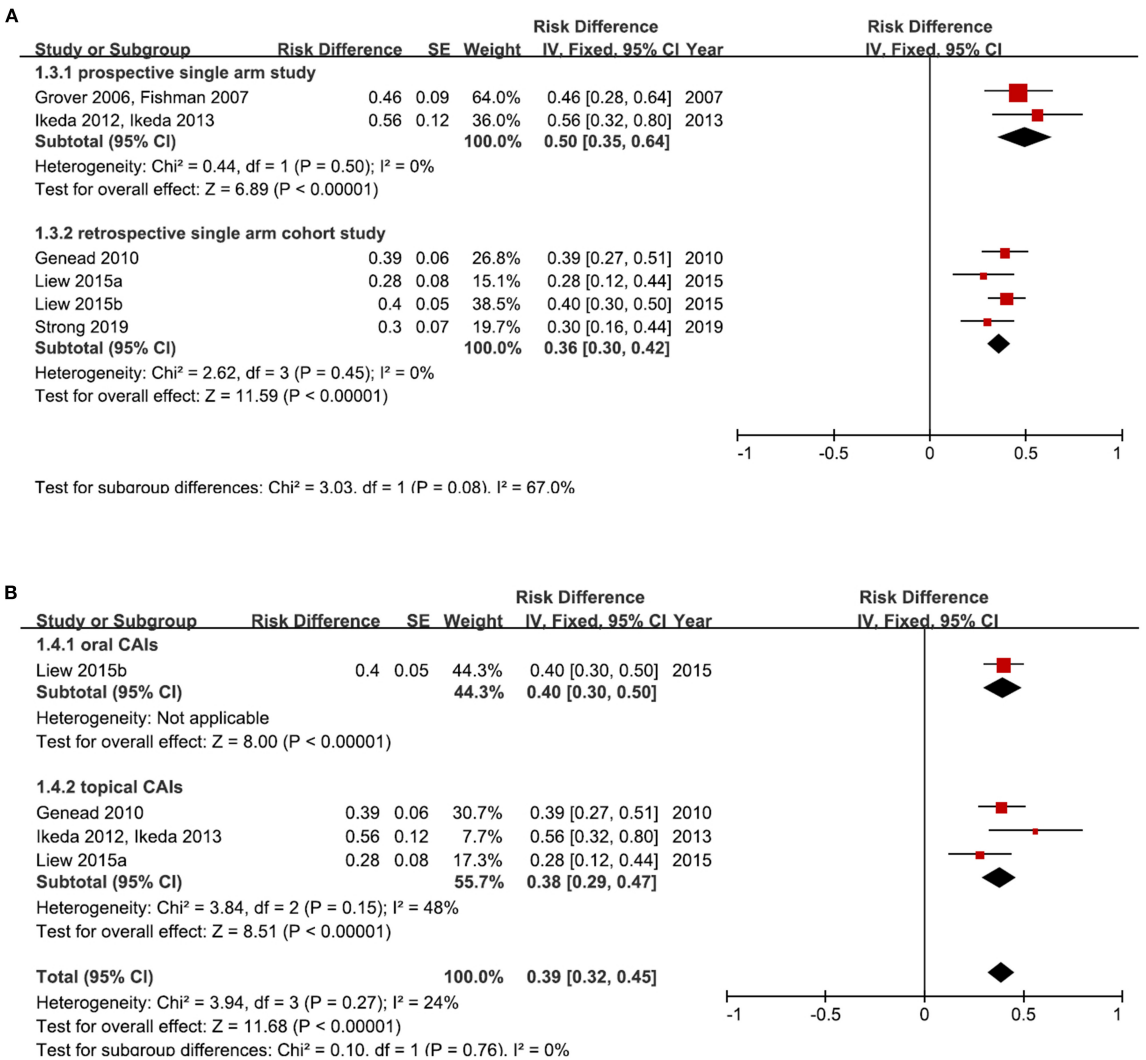
Forest plots for the meta-analysis of the responder proportion after carbonic anhydrase inhibitors (CAIs) treatment**. (A)** Meta-analysis of different study types; **(B)** Subgroup analysis according to different administration methods of CAIs. [Ikeda et al. defined the responder as CMT decreased 20% from baseline. We calculated the 11% decrease of CMT from their published original data; The responder rate of the study Grover et al. (30)/Fishman and Apushkin (7) was calculated from their published original data; Other studies reported the 11% reduction rate directly] [*the study Strong 2019 was used for analysis in **(A)** but not in **(B)** because oral and topical CAIs treatment data cannot be separated in this study. The study Grover et al. (30)/Fishman and Apushkin (7) was used for analysis in **(A)** but not in **(B)** because this study may share some same patients with the study Genead and Fishman (34)].

To be consistent with other studies (7, 17, 34, 36), we define eyes with more than 11% reduction of baseline CMT after treatment as “responders.” The pooled responder proportion for CAIs was 50% in prospective single-arm studies (95% CI: 35%, 64%, I^2^ = 0%) (*n* = 2 studies, 25 patients, 46 eyes), and 36% in retrospective cohort studies (95% CI: 30%, 42%, I^2^ = 0%) (*n* = 3 studies, 138 patients, 254 eyes) (Figure 5A). The responder proportion was 40% for oral CAIs, and 38% for topical CAIs (Figure 5B). The overall responder rate for CAIs was 39% (pooled data from 3 studies, 123 patients, 229 eyes) (Figure 5B). Shimokawa et al. defined eyes with more than 20% reduction of CMT after 1.0% topical dorzolamide treatment as responders, and they reported a higher responder rate of 59.1% and 63.5% in two publications (15, 18).

**Figure 6:**
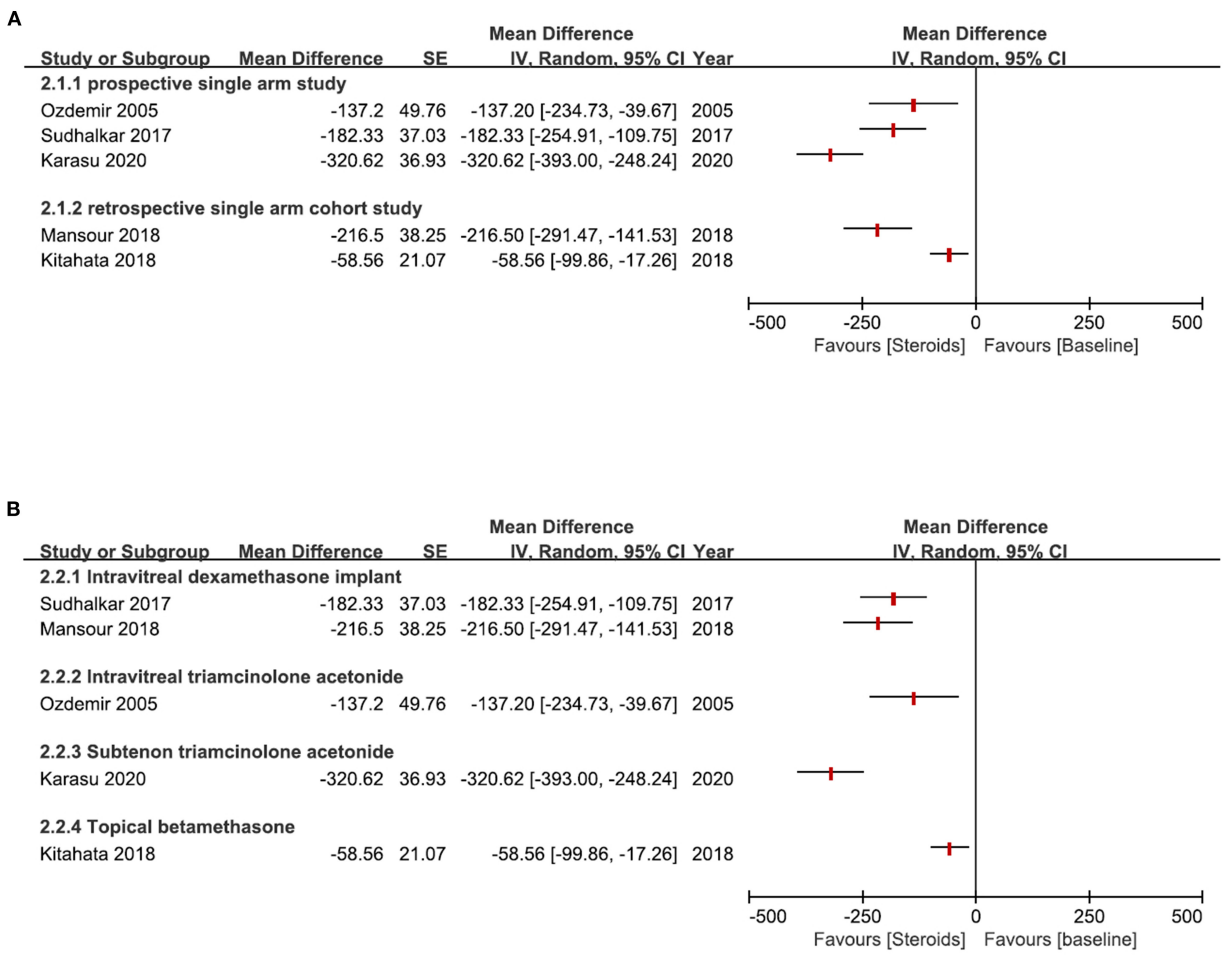
Plots for the change of central macular thickness (CMT) (μm) from baseline after steroids treatment. **(A)** CMT change (μm) in different study types; **(B)** CMT change (μm) of different administration methods of steroids. [for the studies Ozdemir et al. (27) and Sudhalkar et al. (19), the change of CMT was calculated from published original individual data; For the study Kitahata et al. (37), the change of CMT was calculated from the mean/standard deviation data before and after treatment; For the studies Karasu (10) and Mansour et al. (11), the change of CMT was reported in the article].

**Figure 7:**
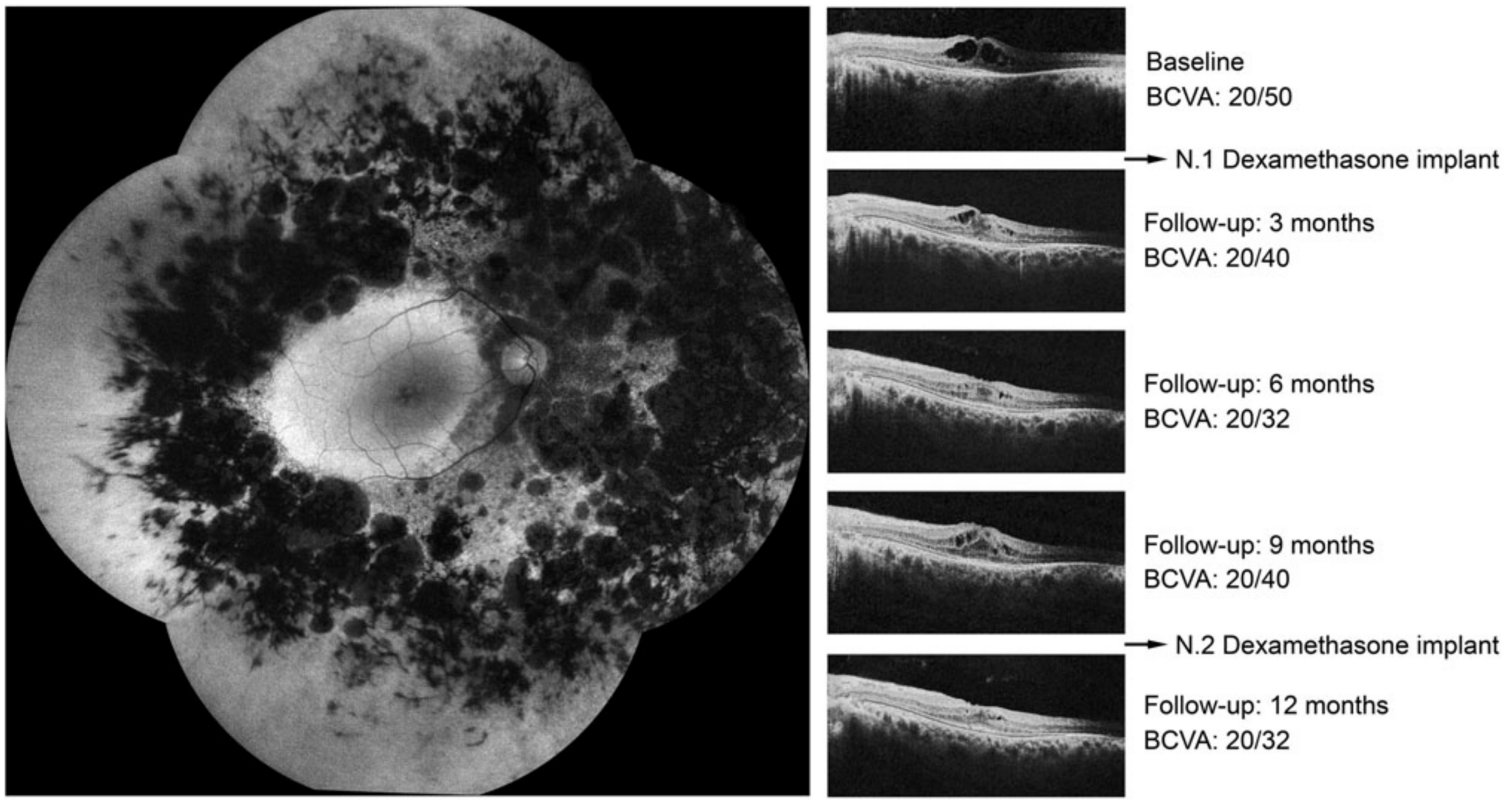
Autofluorescence and optical coherence tomography (OCT) images of a 41-year-old woman affected by macular edema after retinitis pigmentosa (RP) and treated with 1 injection of dexamethasone implant at baseline and at month 9. At baseline, BCVA (Snellen equivalent) was 20/50, and the presence of intraretinal fluid was detected by OCT. At months 3 and 6, BCVA improved, and a reduction in CRT was observed. At month 9, a gradual visual loss and an increase of intraretinal fluid were noted. An additional intravitreal dexamethasone implant was performed at month 9. At 12 months, BCVA improved to 20/32, and no fluid was detected by OCT. This figure was reproduced from Veritti et al. (14). The publisher for this copyrighted material is Mary Ann Liebert, Inc. publishers.

Local steroids were also reported to be useful in treating RP-CME. The average change of CMT varied from −58.56– −320.62 μm after different local steroids treatments, as shown in Figures 6A,B. Moreover, in 2 comparative studies, intravitreal dexamethasone implant (0.7 mg, Ozurdex) showed better results in reducing CMT than the CAIs (14, 16). Figure 7 is a representative of RP-CME treated with dexamethasone implant, illustrating the macular change by OCT during the treatment and follow-up [figure reproduced from Veritti et al. (14)].

The efficacy of anti-VEGF therapy in RP-CME varied among studies. Artunay et al. reported that a single intravitreal injection of ranibizumab (0.5 mg) significantly reduced CMT at 1, 3, 6 months post injection in 15 patients (32). Also, Yuzbasioglu et al. reported a significant reduction of CMT after single or multiple intravitreal injections of bevacizumab (1.25 mg) in 7 patients (13 eyes) (33). However, in a recent study approaching the efficacy of intravitreal aflibercept (2 mg) with a 3+TAE protocol, the treatment failed to achieve a significant overall reduction of CMT, although all the 30 eyes responded after the 1st injection. The responder rate was 37.9% in this study (20).

Regarding other treatments for RP-CME, one session of subliminal micropulse yellow laser treatment was reported to reduce the mean CMT from 651.3 to 247.7 μm in 29 RP patients (32 eyes) at 12 months after the treatment (21). Also, vitrectomy with inner limiting membrane peeling and gas tamponade (40) was reported to reduce the average CMT from 478 to 260 μm in 8 patients (12 eyes) at 6 months post operation. On the other hand, ketorolac eyedrops (41) and lutein supplement (10 or 30 mg/d) (3) failed to decrease the average CMT. Oral minocycline treatment (100 mg bid for 12 m) (NCT02140164) reduced CME in 2 out of 5 patients, and achieved a CMT change of −16.7 ± 42.16 μm and −37.3 ± 52.90 μm at 6 and 12 months (mean ± SD).

### Primary Outcome: Change in BCVA

Improvement or stability of the visual acuity (VA) is the goal of all treatments. All included studies, except a retrospective cohort study (15, 18), have reported VA or BCVA before and after treatment. The reported forms of the change of BCVA after treatment varied among studies, so the data cannot be pooled.

Regarding oral CAIs, acetazolamide (AZM) was the most extensively studied drug to treat RP-CME. In 1988, Cox et al. reported that among 6 RP-CME patients treated with 500 mg/d AZM, 4 had improved VA (5). In 1989, Fishman et al. reported that 10 out of 12 patients had improved VA after AZM treatment (38). Also, Orzalesi et al. reported that among the 5 RP patients who showed leakage in macular on FA, 4 had improved vision after oral AZM therapy (26). However, data from later studies seemed to be less encouraging. In 1998, Moldow et al. observed only small improvements (≤5 ETDRS letters) of VA by AZM treatment (42). Also, Veritti et al. reported an average improvement of only 1.6 ETDRS letters in 30 RP-CME eyes treated with AZM (14). On the other hand, methazolamide has also been used to treat RP-CME. In 1994, Fishman et al. reported a significant VA improvement in a group of 17 patients after methazolamide treatment (50 mg bid). However, an improvement of more than 2 lines (10 ETDRS letters) compared to placebo was only seen in 3 patients (6).

Regarding topical CAIs, Grover et al. reported that 3 out of 15 patients had an improved BCVA of 7 letters or more (Snellen chart) in at least one eye after topical 2% dorzolamide treatment (30). Besides, Genead et al. reported that 10 out of 32 patients (31%) had improved VA by 7 or more letters in at least one eye, and the mean average LogMar VA improved from 0.33 to 0.28 after 2% dorzolamide treatment (34). Also, Reis et al. reported a significant increase in BCVA from baseline at 1, 3, 6 months, but not at 12 months after topical 2% dorzolamide treatment, with 7 eyes (54%) had an improvement of 7 letters or more (Snellen chart) (41). However, in 3 other studies, topical dorzolamide treatment failed to achieve a significant improvement in VA (16, 35, 39, 43).

Different treatments of steroids also showed varied effects in VA improvement, among which intravitreal dexamethasone implant (0.7 mg, Ozurdex) seemed promising. Sudhalkar et al. reported a significant improvement of corrected VA in 5 patients underwent dexamethasone implant treatment (19). The result was further confirmed by a later study including 45 eyes from 34 patients, which observed an improvement of mean BCVA from 0.61 to 0.37 (*P* = 0.012) (11). Furthermore, two studies published in 2020 reported that intravitreal dexamethasone implant was superior to CAIs in BCVA improvement (14, 16).

Oral deflazacort was evaluated in one study, with the near BCVA improved significantly (*p* < 0.01) while the far BCVA improved slightly (*p* < 0.05) (9). Intravitreal triamcinolone acetonide (IVTA) (4 mg) was reported to transiently improve VA in 2 out of 5 patients at 1 month after injection, but not at 3 and 6 months post injection (27). While in another study, no significant change of BCVA was observed over a 12 months period after IVTA (4 mg) (31). However, subtenon TA (40 mg) was reported to improve VA in all participants, with a change of LogMAR BCVA from 1.09 at baseline to 0.54 at 3 months post injection (10). Last, topical 0.1% betamethasone treatment failed to improve BCVA in RP-CME (37).

A single dose of intravitreal ranibizumab (0.5 mg) was reported to improve BCVA in 9 out of 15 treated eyes, however, the mean BCVA change was not significantly different between ranibizumab and control group (32). In the meantime, intravitreal bevacizumab (1.25 mg) was reported to improve VA from 5/400–20/100 to 20/200–20/63 in 13 treated eyes (33). However, intravitreal aflibercept (2 mg) administrated with a 3+TAE protocol failed to show any help in BCVA (20).

Regarding LASER treatment, Newsome et al. reported an improvement of VA in 6 out of 16 eyes treated with grid photocoagulation. Authors also found this method to be effective in preventing worsening of VA (25). On the other hand, subliminal micropulse yellow laser was reported to improve median BCVA from 66.8 ETDRS letters to 70.0 letters (*p* = 0.18), with subjective improvement of central vision, color vision and contrast sensitivity in 68% of patients (21).

In 2003, Garci'a-Arumi' et al. reported that PPV improved the mean VA from 20/115 to 20/45. The VA improved in 10 out of 12 treated eyes, with an average improvement of 3 lines (ETDRS chart, *p* = 0.028) (40). Meanwhile, Reis et al. reported that ketorolac eyedrops improved LogMAR BCVA from 0.37 ± 0.17 at baseline to 0.27 ± 0.18 at 6 months (*p* = 0.03), and to 0.28 ± 0.16 at 12 months (*p* = 0.02) (41). Lutein supplement and oral minocycline didn't show any help in VA (3).

### Secondary Outcome: Change in FA Leakage

Evaluation of CME by FA leakage is subjective to some extent, however, in the pre-OCT era, assessment of FA leakage in the macular area provided useful information about macular edema. Overall, 10 of our included studies reported changes in FA leakage after treatment, with different criteria to grade the leakage. Seven of these studies approached the CAIs treatment, 5 of which studied the efficacy of AZM on RP-CME. The ratio of reduced FA leakage in the macular region was reported to be 33% (2 out of 6 patients) (5), 50% (6 out of 12 patients) (38), 0% (0 out of 5) (26), 43% (3 out of 7) (42) and 20% (1 out of 5) (28) after AZM treatment. Meanwhile, methazolamide was reported to decrease FA leakage in 9 out of 17 patients (6), and topical dorzolamide treatment reduced leakage in 2 out of 5 patients (43).

In 1987, Newsome et al. reported that grid photocoagulation reduced FA leakage in the macular area in 13 out of 16 treated patients (25). In 2002, Giusti et al. reported that oral deflazacort reduced FA macular leakage in 47% of participants at the study end (12 m) (9). And vitrectomy was reported to reduce macular leakage in 75% of patients (40).

### Secondary Outcome: Rebound of CME

Rebound of CME was not rare in spite of continual use of medications. Apushkin et al. reported that 3 out of 6 patients had recurrent CME during prolonged treatment of AZM for 8–12 weeks (29). Also, Fishman et al. reported that all 3 patients that had oral methazolamide (50 mg bid) for prolonged 6–12 weeks experienced rebound of CME (44). For topical dorzolamide, the recurrence rate was reported to be 27% (4 out of 15 patients) (30), 28% (5 in 18 eyes) (39), and 35% (14 in 40 patients) (18). Regarding steroids treatment, Giusti et al. reported that oral deflazacort tapering therapy resulted in reduction of FA leakage in 100% patients at 4 months, while the reduction was observed in only 47% patients at 12 months compared to baseline (9). For IVTA (27), subtenon TA (10) and intravitreal dexamethasone implant treatments (11, 16, 19), multiple injections were needed because of recurrence of CME. Repeated injections were also common in anti-VEGF therapy for RP-CME (20, 33).

### Secondary Outcome: Adverse Effects

Among the included studies, adverse effects were reported in 14 studies, and were reported as not present in 13 studies, and were not mentioned in other studies. The side effects of oral AZM and methazolamide included tingling of the extremities, gastrointestinal tract upset, fatigue, dizziness and altered taste sensation (6, 29, 38, 42). Also, Veritti et al. reported that one patient developed aciduria and one patient developed kidney stones after continual use of AZM for 9 and 11 months, respectively (14). Besides, dorzolamide eyedrops was reported to cause a burning and stinging sensation right after administration (30, 43).

No side effects were reported for oral deflazacort, however, regarding local steroids, elevation of intraocular pressure (IOP) and cataract formation was reported (9–11, 16, 19, 37). On the other hand, in the 7 patients treated with oral minocycline (100 mg bid for 12 m), 15 adverse events (3 ocular and 12 non-ocular) were recorded within 16 months after the beginning of treatment (NCT02140164). No adverse effects were reported in anti-VEGF therapy, LASER therapy, PPV, ketorolac eyedrops and lutein supplement for the treatment of RP-CME (3, 20, 21, 25, 32, 33, 40, 41).

## Discussion

In the present study, we systematically review the existing treatments for RP-CME on the aspects of efficacy and safety. From the pooled data, we found that CAIs (including oral and topical CAIs) significantly decreased CMT, with the mean change of −45.64 μm. And the responder proportion was 39% (reduction >11% initial CMT). Multiple mechanisms are implicated in the therapeutic effects of the CAIs in RP-CME. Moldow et al. reported that AZM decreased passive permeability and stimulated unidirectional permeability for fluorescein in the retina of 7 RP-CME patients (42), and they pointed out that AZM reduced retinal vascular leakage and increased active transport through the BRB. In animal models, AZM was demonstrated to accelerate subretinal fluid absorption and promote the adhesion between neuroretina and pigment epithelium, and this effect was attributed to the influence on the carbonic anhydrases located at both apical and basal surfaces of the RPE (1, 45, 46). Regarding spatial distribution of CME, Strong et al. revealed that all RP-CME had fluid in the inner nuclear layer (INL), while all responders to CAIs had coexisting fluid in the outer nuclear layer (ONL). However, not all patients presented with coexisting INL and ONL fluid responded to CAIs (17). They also found that epiretinal membranes had minimal influence on drug efficacy, possibly because the CAIs had better access to the basal surface of the RPE than the neuroretina (17).

Carbonic anhydrases (CA) are ubiquitously distributed in multiple organs and tissues and are involved in various physiological processes (47). CAIs have been used clinically to treat epilepsy, obesity, glaucoma, altitude sickness, idiopathic intracranial hypertension, and some tumors (48). AZM and methazolamide are non-selective CA inhibitors, thus when administrated systematically, they bring about side effects (47), which restricted the long-term use of these drugs. Moreover, it's noteworthy that drug allergy occurs in ~7.4% of patients exposed to sulfonamide antibiotics (49). CAIs are non-antimicrobial sulfonamides (50). Although cross-reaction between antimicrobial and non-antimicrobial sulfonamides is still controversial (50), application of CAIs in individuals with history of sulfonamide allergy is not recommended, especially when other therapeutic options exist.

Steroids were employed in the treatment of RP-CME on the basis that inflammatory responses were implicated in the pathogenesis. In 1988, Newsome et al. detected the presence of various subsets of T lymphocytes including T helper, T suppressor lymphocytes and natural killer cells from the vitreous sample of RP patients (51). Besides, Yoshida et al. observed inflammatory cells in the anterior vitreous cavity of 37.3% RP patients. Also, they found the levels of proinflammatory cytokines were increased in both the aqueous and vitreous samples of RP patients compared to the control (52). Moreover, Heckenlively et al. detected the existence of serum antiretinal protein antibodies in 27 out of 30 RP patients with macular edema, but only in 4 out of 30 RP patients without macular edema (53), indicative of the implication of inflammation process in RP-CME. Current evidence demonstrated that steroids were beneficial in the management of RP-CME, with the average change of CMT from baseline varied from −58.56 to −320.62μm by different steroids treatments (10, 11, 19, 27, 37). In the 2 studies compared the treatment of intravitreal dexamethasone implant to CAIs (AZM and topical dorzolamide, respectively), the dexamethasone implant was reported to be more effective in reducing CMT as well as improving VA (14, 16). However, the risk of increased IOP and the development of cataract need to be considered, and aphakic or pseudophakic eyes may benefit more from local steroids. Although no side effects of oral deflazacort were observed during a 12-months period (9), we don't recommend oral steroids for RP-CME because of potential risk of infection and adrenal crisis associated with systematic steroids (54).

The rationale for anti-VEGF therapy in RP-CME is still debatable. VEGF has been identified as a neuroprotective factor, which plays an important role in neuron survival and in the functional maintenance of retinal ganglion cells, photoreceptors and Muller cells (55, 56). Salom et al. reported that the aqueous level of VEGFA was 94.9 ± 99.8 pg/ml in eyes of RP patients, and 336.5 ± 116.8 pg/ml in control eyes (p <0.001). They speculated that the inadequate VEGF level may contribute to the degeneration of retinal vasculature in RP patients, and questioned the validity of anti-VEGF therapy in RP-CME (56, 57). In the present systematic review, 3 included studies approached the efficacy of different anti-VEGF reagents in RP-CME. Ranibizumab significantly reduced mean CMT in 15 included eyes, but the BCVA improvement was not significant compared to control (32). Bevacizumab was reported to be effective in reducing CMT as well as improving BCVA in 7 patients (33). Aflibercept failed to reduce mean CMT or improve BCVA in a group of 30 patients, in spite of an initial response after the first injection in all patients (20). Based on existing evidence, anti-VEGF therapy may not be suitable for treating RP-CME.

Grid photocoagulation was reported to reduce FA leakage in 13 out of 16 treated eyes and improve VA in 6 out of the 16 eyes (25). However, due to possible deterioration of the severely constricted visual fields in RP patients after treatment, the validity of grid photocoagulation is questionable (58). In contrast, micropulse LASER may be more suitable in treating RP-CME. The length of each LASER pulse is 100–300 μs, so that the RPE cells are only stimulated, instead of being destroyed by thermal heat (59). The stimulation of RPE cells induces an altered profile of gene expression, which is beneficial for tissue healing and repair of the inner BRB (59). Micropulse LASER was reported to reduce CMT significantly in RP-CME. Although the change in BCVA was not significant, 86% of participants had subjective improvement of vision (21). Possessing the non-invasive, safe and repeatable properties, micropulse LASER treatment may be promising in the treatment of RP-CME. However, more clinical studies are needed to verify the efficacy and safety of this treatment approach.

Vitrectomy was employed to treat RP-CME on the hypothesis that vitreous traction played a role in the pathogenesis. Vitrectomy with internal limiting membrane removal and gas tamponade was reported to reduce CMT and significantly improve VA in 12 RP-CME eyes (40). However, this is the only study approached the efficacy and safety of vitrectomy in RP-CME, with small number of patients. Concerning its invasive nature and the potential risk of complications, vitrectomy should not be considered when other methods are effective and available.

It is noteworthy that although some of our included studies reported the CMT reduction along with the significant improvement of BCVA (10, 11, 14, 16, 19, 40), several other included studies reported that the remarkable reduction of CMT accompanied only minimal improvement of BCVA (17, 27, 28, 32, 39). Chung et al. attributed this to the irreversible photoreceptor cell loss and permanent functional damage due to chronic macular edema and the genetic degenerative nature of the photoreceptor cells in RP patients (28). Two recent studies found that the duration of CME did not affect the positive anatomical change after treatment (13, 20). Nevertheless, Strong et al. pointed out that the intactness of the photoreceptor layer and the ellipsoid zone within the macular was important for the improvement of VA after treatment (20). Thus, early management of CME may be vital in preserving the vision in RP patients.

Rebound of RP-CME was reported in some treatment approaches including subtenon TA, intravitreal TA and intravitreal dexamethasone implant (10, 11, 16, 19, 27, 31). In these studies, patients responded well to retreatments, indicating that the rebound was due to drug elimination. However, in several included studies, rebound of RP-CME was observed in spite of continual use of the drugs (AZM, methazolamide and dorzolamide included) (29, 30, 39, 44). Although rebound of CME might be partially attributed to poor patient compliance (18, 44), the underlying mechanisms were unknown. A recent study found that the high baseline CMT value was significantly associated with recurrence of CME in topical dorzolamide treatment (18). Authors also reported that in rebound RP-CME under dorzolamide treatment, additional topical steroids was useful to reduce CMT (18).

The most common measurements for evaluating RP-CME include BCVA, CMT measured by OCT, and FA leakage. Chung et al. reported that among the included 10 patients who had macular cyst change in OCT, 5 had fluorescein leakage in FA (28). OCT is more sensitive in detecting RP-CME, because OCT detects the fluid accumulation both from RPE pumping dysfunction and BRB breakdown, while FA only detects the latter (57). Moreover, inconsistency between OCT and FA may also rise from that FA detects the real-time vascular or RPE leakage and the accumulation of dye during the examination, while OCT detects the intraretinal fluid accumulating from a relatively long period of time. On the other hand, the macular sensitivity detected by Humphrey field analyzer 10–2 program may also be helpful in evaluating the visual function change in RP-CME (35).

Compared with the previous systematic reviews (4, 8), our study included meta-analyses in order to evaluate the extent of the change of CMT after CAIs treatment. While compared with the previous published meta-analysis (12), our study added data from recently published studies, as well as calculated the pooled responder rate. However, the current study has limitations. Most of our included studies had small patient number, due to the relatively low incidence of RP-CME. The 6 RCTs included in our study had a patient number of 5–39, and 5 of the RCTs were crossover designed studies. More than half of the included studies lacked a control group. Moreover, the follow-up period of all included prospective studies were no more than 2 years, which is insufficient regarding the refractory and recurrent nature of RP-CME.

Another limitation of our study was the heterogeneity among the included trials, which restricted the pooling of data. For example, some of the included studies measured visual acuity (VA) as the therapeutic outcome (27, 33), while other studies measured the best corrected visual acuity (BCVA), which was more accurate for evaluating the visual function. Also, the grading system for FA leakage was different among studies (5, 28, 38). Moreover, the measurement of CMT was not consistent among the studies we used for meta-analysis. Some of the studies measured the foveal thickness (FT) (28, 37), while some other studies measured the central subfield thickness (CST) or foveal zone thickness (FZT) which was defined as the mean thickness of the central 1,000 μm diameter area of the macula (11, 34, 35, 39). Some studies didn't mention their definition of CMT at all (10, 17, 19, 27, 36), while some other studies reported both FT and FZT (for these studies we used FZT values for analyzing) (7, 29, 30). The inconsistency in CMT measurement decreased the accuracy of our data pooling.

Last but not the least, in most of our included studies, RP was diagnosed by typical clinical signs and symptoms (night-blindness, restricted visual field, pale optic disc, retinal vessel attenuation and bone spicule pigments) as well as the change of electroretinography. Nevertheless, genetic mutation test was not routinely carried out, even in recent years. Twelve of the included studies recorded the inheritance pattern of RP (autosomal dominant, autosomal recessive, X-linked recessive or sporadic) (5, 9–11, 15, 20, 30, 34, 36, 37, 40, 43), while only 3 studies reported the specific mutation of genes (20, 30, 34). Liew et al. reported that the macular edema of autosomal recessive RP responded better to topical dorzolamide than autosomal dominant RP (36). However, Strong et al. found no association between inheritance pattern and response to intravitreal aflibercept (20). As diverse genetic types of RP may respond differently to therapy, genetic tests are recommended in future studies, which will add to our knowledge of RP-CME.

Because of the limitations mentioned above, no high-quality evidence could be provided based on existing reports. More controlled clinical trials are needed in future, since single-arm studies cannot rule out the influence of natural progression of the disease. Topical CAIs, local steroids, topical NSAIDS and micropulse LASER are worthwhile for more clinical trials as the side effects of these treatments are milder compared to oral CAIs and systematic steroids. Thus, these treatments may be used for a relatively long period, or can be repeated (retreated). On the other hand, standard measurements, for example, BCVA (in logMAR or in ETDRS letters) and CST (the average thickness of the central 1,000 μm diameter area of the macular) are recommended in future studies.

To sum up, topical CAIs, oral CAIs and local steroids were proved to be effective in treating RP-CME. However, due to the overall inferior design and small patient number of the included studies, the grade of evidence was very low. Systematic steroids, LASER, NSAIDS and PPV may also be effective, nevertheless, considering the limited number of studies, no conclusion could be drawn regarding these treatments. More well-designed and conducted studies, especially RCTs, are desperately needed.

## Data Availability Statement

The original data are presented in the article and online supplementary files. Further inquiries can be directed to the corresponding author XP ( 74000041@ccmu.edu.cn) or to CC ( chenchenmd@aliyun.com).

## Author Contributions

XP and CC conceived and designed the project. CC and XL performed the literature search, data extraction, study quality assessment, and data analysis. CC drafted the manuscript. XP critically revised the paper. All authors commented on previous versions of the manuscript, and read and approved the final manuscript.

## Funding

This work was supported by The Capital Health Research and Development of Special (No. SF-2018-2-1081), Capital Medical University Affiliated Beijing Tongren Hospital Key Medical Development Plan (trzdyxzy201801). This work was also supported by National Natural Science Foundation of China (No. 81660167); Personnel project funded by Health Commission of Yunnan Province (H-2019057) and Research Grant ZX2019-02-01, YXZX-05 from the Yunnan Clinical Medicine Center for Ocular Disease.

## Conflict of Interest

The authors declare that the research was conducted in the absence of any commercial or financial relationships that could be construed as a potential conflict of interest.

## Publisher's Note

All claims expressed in this article are solely those of the authors and do not necessarily represent those of their affiliated organizations, or those of the publisher, the editors and the reviewers. Any product that may be evaluated in this article, or claim that may be made by its manufacturer, is not guaranteed or endorsed by the publisher.

## References

[1] Huckfeldt RM. Comander J. Management of cystoid macular edema in retinitis pigmentosa. Semin Ophthalmol. (2017) 32:43–51. 10.1080/08820538.2016.1228404 27748628

[2] Amato A Arrigo A Aragona E Manitto MP Saladino A Bandello F . Gene therapy in inherited retinal diseases: an update on current state of the art. Front Med. (2021) 8:750586. 10.3389/fmed.2021.750586 34722588 PMC8553993

[3] Adackapara CA Sunness JS Dibernardo CW Melia BM Dagnelie G. Prevalence of cystoid macular edema and stability in oct retinal thickness in eyes with retinitis pigmentosa during a 48-week lutein trial. Retina. (2008) 28:103–10. 10.1097/IAE.0b013e31809862aa 18185146

[4] Strong S Liew G Michaelides M. Retinitis pigmentosa-associated cystoid macular oedema: pathogenesis and avenues of intervention. Br J Ophthalmol. (2017) 101:31–7. 10.1136/bjophthalmol-2016-309376 27913439 PMC5256121

[5] Cox SN Hay E Bird CA. Treatment of chronic macular edema with acetazolamide. Arch Ophthalmol. (1988) 106:1190–5. 10.1001/archopht.1988.01060140350030 3415543

[6] Fishman GA Gilbert LD Anderson RJ Marmor MF Weleber RG Viana AM. Effect of methazolamide on chronic macular edema in patients with retinitis pigmentosa. Ophthalmology. (1994) 101:687–93. 10.1016/S0161-6420(94)31277-2 8152764

[7] Fishman GA Apushkin AM. Continued use of dorzolamide for the treatment of cystoid macular oedema in patients with retinitis pigmentosa. Br J Ophthalmol. (2007) 91:743–5. 10.1136/bjo.2006.107466 17215269 PMC1955610

[8] Bakthavatchalam M Lai FHP Rong SS Ng DS Brelen EM. Treatment of cystoid macular edema secondary to retinitis pigmentosa: a systematic review. Surv Ophthalmol. (2018) 63:329–39. 10.1016/j.survophthal.2017.09.009 28987613

[9] Giusti C Forte R Vingolo ME. Deflazacort treatment of cystoid macular edema in patients affected by retinitis pigmentosa: a pilot study. Eur Rev Med Pharmacol Sci. (2002) 6:1–8 12608650

[10] Karasu B. Short-term outcomes of subtenon triamcinolone acetonide injections in patients with retinitis pigmentosa-associated cystoid macular edema unresponsive to carbonic anhydrase inhibitors. Int Ophthalmol. (2020) 40:677–87. 10.1007/s10792-019-01228-z 31773389

[11] Mansour AM Sheheitli H Kucukerdonmez C Sisk RA Moura R Moschos MM . Intravitreal dexamethasone implant in retinitis pigmentosa-related cystoid macular edema. Retina. (2018) 38:416–23. 10.1097/IAE.0000000000001542 28221257

[12] Huang Q Chen R Lin X Xiang Z. Efficacy of carbonic anhydrase inhibitors in management of cystoid macular edema in retinitis pigmentosa: a meta-analysis. PLoS ONE. (2017) 12:e0186180. 10.1371/journal.pone.0186180 29023491 PMC5638411

[13] Park UC Park JH Yoon CK Yu GH. Microstructural changes in cystoid macular edema in retinitis pigmentosa after intravitreal dexamethasone implant injection. Retina. (2021) 41:852–60. 10.1097/IAE.0000000000002944 32796442

[14] Veritti D Sarao V De Nadai K Chizzolini M Parmeggiani F Perissin L . dexamethasone implant produces better outcomes than oral acetazolamide in patients with cystoid macular edema secondary to retinitis pigmentosa. J Ocul Pharmacol Ther. (2020) 36:190–7. 10.1089/jop.2018.0153 31886707

[15] Shimokawa S Fujiwara K Murakami Y Funatsu J Nakatake S Yoshida N . Effect of topical dorzolamide on cystoid macular edema in retinitis pigmentosa. Ophthalmol Retina. (2020) 4:1036–9. 10.1016/j.oret.2020.05.012 32454228

[16] Park UC Park JH Ma DJ Cho IH Oh BL Yu G. A randomized paired-eye trial of intravitreal dexamethasone implant for cystoid macular edema in retinitis pigmentos. Retina. (2020) 40:1359–66. 10.1097/IAE.0000000000002589 31166248

[17] Strong SA Hirji N Quartilho A Kalitzeos A Michaelides M. Retrospective cohort study exploring whether an association exists between spatial distribution of cystoid spaces in cystoid macular oedema secondary to retinitis pigmentosa and response to treatment with carbonic anhydrase inhibitors. Br J Ophthalmol. (2019) 103:233–7. 10.1136/bjophthalmol-2017-311392 29706600

[18] Shimokawa S Murakami Y Fujiwara K Funatsu J Nakatake S Koyanagi Y . Recurrence rate of cystoid macular edema with topical dorzolamide treatment and its risk factors in retinitis pigmentosa. Retina. (2021) 62:3287. 10.1097/IAE.0000000000003286 34393209

[19] Sudhalkar A Kodjikian L Borse N. Intravitreal dexamethasone implant for recalcitrant cystoid macular edema secondary to retinitis pigmentosa: a pilot study. Graefes Arch Clin Exp Ophthalmol. (2017) 255:1369–74. 10.1007/s00417-017-3660-7 28378252

[20] Strong SA Peto T Bunce C Xing W Georgiou M Esposti SD . Prospective exploratory study to assess the safety and efficacy of aflibercept in cystoid macular oedema associated with retinitis pigmentosa. Br J Ophthalmol. (2020) 104:1203–8. 10.1136/bjophthalmol-2019-315152 32041720 PMC7577098

[21] Arslan U. Management of cystoid macular edema secondary to retinitis pigmentosa via subliminal micropulse yellow laser. Lasers Med Sci. (2021) 36:317–23. 10.1007/s10103-020-03031-0 32363437

[22] Page MJ McKenzie JE Bossuyt PM Boutron I Hoffmann TC Mulrow CD . The PRISMA 2020 statement: an updated guideline for reporting systematic reviews. Syst Rev. (2021) 10:89. 10.1186/s13643-021-01626-4 33781348 PMC8008539

[23] Sterne JAC Savovic J Page MJ Elbers RG Blencowe NS Boutron I . RoB 2: a revised tool for assessing risk of bias in randomised trials. BMJ. (2019) 366:l4898. 10.1136/bmj.l4898 31462531

[24] Slim K Nini E Forestier D Kwiatkowski F Panis Y Chipponi J. Methodological index for non-randomized studies (minors): development and validation of a new instrument. ANZ J Surg. (2003) 73:712–6. 10.1046/j.1445-2197.2003.02748.x 12956787

[25] Newsome DA Blacharski AP. Grid photocoagulation for macular edema in patients with retinitis pigmentosa. Am J Ophthalmol. (1987) 103:161–6. 10.1016/S0002-9394(14)74221-7 3812618

[26] Orzalesi N Pierrottet C Porta A Aschero M. Long-term treatment of retinitis pigmentosa with acetazolamide. A pilot study. Graefes Arch Clin Exp Ophthalmol. (1993) 231:254–6. 10.1007/BF00919100 8319913

[27] Ozdemir H Karacorlu M Karacorlu S. Intravitreal triamcinolone acetonide for treatment of cystoid macular oedema in patients with retinitis pigmentosa. Acta Ophthalmol Scand. (2005) 83:248-51. 10.1111/j.1600-0420.2005.00395.x 15799743

[28] Chung H Hwang JU Kim JG Yoon HY. Optical coherence tomography in the diagnosis and monitoring of cystoid macular edema in patients with retinitis pigmentosa. Retina. (2006) 26:922–7. 10.1097/01.iae.0000250008.83779.23 17031294

[29] Apushkin MA Fishman GA Grover S Janowicz JM. Rebound of cystoid macular edema with continued use of acetazolamide in patients with retinitis pigmentosa. Retina. (2007) 27:1112–8. 10.1097/IAE.0b013e31805f6b79 18040255

[30] Grover S Apushkin MA Fishman AG. Topical dorzolamide for the treatment of cystoid macular edema in patients with retinitis pigmentosa. Am J Ophthalmol. (2006) 141:850–8. 10.1016/j.ajo.2005.12.030 16546110

[31] Scorolli L Morara M Meduri A Reggiani LB Ferreri G Scalinci SZ . Treatment of cystoid macular edema in retinitis pigmentosa with intravitreal triamcinolone. Arch Ophthalmol. (2007) 125:759–64. 10.1001/archopht.125.6.759 17562986

[32] Artunay O Yuzbasioglu E Rasier R Sengul A Bahcecioglu H. Intravitreal ranibizumab in the treatment of cystoid macular edema associated with retinitis pigmentosa. J Ocul Pharmacol Ther. (2009) 25:545–50. 10.1089/jop.2009.0089 20028262

[33] Yuzbasioglu E Artunay O Rasier R Sengul A Bahcecioglu H. Intravitreal bevacizumab (Avastin) injection in retinitis pigmentosa. Curr Eye Res. (2009) 34:231–7. 10.1080/02713680802710692 19274531

[34] Genead MA Fishman AG. Efficacy of sustained topical dorzolamide therapy for cystic macular lesions in patients with retinitis pigmentosa and usher syndrome. Arch Ophthalmol. (2010) 128:1146–50. 10.1001/archophthalmol.2010.172 20837798 PMC3696579

[35] Ikeda Y Yoshida N Notomi S Murakami Y Hisatomi T Enaida H . Therapeutic effect of prolonged treatment with topical dorzolamide for cystoid macular oedema in patients with retinitis pigmentosa. Br J Ophthalmol. (2013) 97:1187–91. 10.1136/bjophthalmol-2012-303005 23782868

[36] Liew G Moore AT Webster AR Michaelides M. Efficacy and prognostic factors of response to carbonic anhydrase inhibitors in management of cystoid macular edema in retinitis pigmentosa. Invest Ophthalmol Vis Sci. (2015) 56:1531–6. 10.1167/iovs.14-15995 25670491

[37] Kitahata S Hirami Y Takagi S Kime C Fujihara M Kurimoto Y . Efficacy of additional topical betamethasone in persistent cystoid macular oedema after carbonic anhydrase inhibitor treatments in retinitis pigmentosa. BMJ Open Ophthalmol. (2018) 3:e000107. 10.1136/bmjophth-2017-000107 29657976 PMC5895969

[38] Fishman GA Gilbert LD Fiscella RG Kimura AE Jampol ML. Acetazolamide for treatment of chronic macular edema in retinitis pigmentosa. Arch Ophthalmol. (1989) 107:1445–52. 10.1001/archopht.1989.01070020519031 2803090

[39] Ikeda Y Hisatomi T Yoshida N Notomi S Murakami Y Enaida H . The clinical efficacy of a topical dorzolamide in the management of cystoid macular edema in patients with retinitis pigmentosa. Graefes Arch Clin Exp Ophthalmol. (2012) 250:809–14. 10.1007/s00417-011-1904-5 22215259

[40] García-Arumí J Martinez V Sararols L Corcostegui B. Vitreoretinal surgery for cystoid macular edema associated with retinitis pigmentosa. Ophthalmology. (2003) 110:1164–9. 10.1016/S0161-6420(03)00259-8 12799242

[41] Reis RFL Moreira-Gonçalves N Silva SEE Brandão EM Falcão-Reis MF. Comparison of topical dorzolamide and ketorolac treatment for cystoid macular edema in retinitis pigmentosa and usher's syndrome. Ophthalmologica. (2015)233:43–50. 10.1159/000368052 25428176

[42] Moldow B Sander B Larsen M Engler C Li B Rosenberg T . The effect of acetazolamide on passive and active transport of fluorescein across the blood-retina barrier in retinitis pigmentosa complicated by macular oedema. Graefes Arch Clin Exp Ophthalmol. (1998) 236:881–9. 10.1007/s004170050175 9865617

[43] Grover S Fishman GA Fiscella RG Adelman EA. Efficacy of dorzolamide hydrochloride in the management of chronic cystoid macular edema in patients with retinitis pigmentosa. Retina. (1997) 17:222–31. 10.1097/00006982-199705000-00009 9196934

[44] Fishman GA Glenn AM Gilbert DL. Rebound of macular edema with continued use of methazolamide in patients with retinitis pigmentosa. Arch Ophthalmol. (1993) 111:1640–6. 10.1001/archopht.1993.01090120062023 8155034

[45] Marmor MF Maack T. Enhancement of retinal adhesion and subretinal fluid resorption by acetazolamide. Invest Ophthalmol Vis Sci. (1982) 23:121–4. 7085214

[46] Marmor MF Negi A. Pharmacologic modification of subretinal fluid absorption in the rabbit eye. Arch Ophthalmol. (1986) 104:1674–7. 10.1001/archopht.1986.01050230112043 3778286

[47] Aggarwal M McKenna R. Update on carbonic anhydrase inhibitors: a patent review (2008 - 2011). Expert Opin Ther Pat. (2012) 22:903–15. 10.1517/13543776.2012.707646 22788994

[48] Van Berkel MA Elefritz JL. Evaluating off-label uses of acetazolamide. Am J Health Syst Pharm. (2018) 75:524–31. 10.2146/ajhp170279 29626002

[49] Zhou L Dhopeshwarkar N Blumenthal KG Goss F Topaz M Slight SP . Drug allergies documented in electronic health records of a large healthcare system. Allergy. (2016) 71:1305–13. 10.1111/all.12881 26970431 PMC12841114

[50] Kelly TE Hackett HP. Acetazolamide and sulfonamide allergy: a not so simple story. High Alt Med Biol. (2010) 11:319–23. 10.1089/ham.2010.1051 21190500

[51] Newsome DA Michels GR. Detection of lymphocytes in the vitreous gel of patients with retinitis pigmentosa. Am J Ophthalmol. (1988) 105:596–602. 10.1016/0002-9394(88)90050-5 3377040

[52] Yoshida N Ikeda Y Notomi S Ishikawa K Murakami Y Hisatomi T . Clinical evidence of sustained chronic inflammatory reaction in retinitis pigmentosa. Ophthalmology. (2013) 120:100–5. 10.1016/j.ophtha.2012.07.006 22986109

[53] Heckenlively JR Aptsiauri N Nusinowitz S Peng C Hargrave AP. Investigations of antiretinal antibodies in pigmentary retinopathy and other retinal degenerations. Trans Am Ophthalmol Soc. (1996) 94:179–200; discussion 200–6. 8981696 PMC1312095

[54] Grennan D Wang S. Steroid side effects. JAMA. (2019) 322:282 10.1001/jama.2019.8506 31310300

[55] Parodi MB Iacono P Da Pozzo S. Anti-VEGF and retinal dystrophies. Curr Drug Targets. (2020) 21:1201–7. 10.2174/1389450121666200428103334 32342816

[56] Salom D Diaz-Llopis M Garcia-Delpech S Udaondo P Sancho-Tello M Romero JF. Aqueous humor levels of vascular endothelial growth factor in retinitis pigmentosa. Invest Ophthalmol Vis Sci. (2008) 49:3499–502. 10.1167/iovs.07-1168 18326689

[57] Salom D Diaz-Llopis M Garcia-Delpech S Udaondo P Romero FJ Millan JM . Intravitreal ranibizumab in the treatment of cystoid macular edema associated with retinitis pigmentosa. J Ocul Pharmacol Ther. (2010) 26:531–2. 10.1089/jop.2010.0044 20925582

[58] Heckenlively JR. Grid photocoagulation for macular edema in patients with retinitis pigmentosa. Am J Ophthalmol. (1987) 104:94–5. 10.1016/0002-9394(87)90308-4 3605293

[59] Scholz P Altay L Fauser S. A review of subthreshold micropulse laser for treatment of macular disorders. Adv Ther. (2017) 34:1528–55. 10.1007/s12325-017-0559-y 28540655 PMC5504253

