# Supplementary material for: Management of Cystoid Macular Edema in Retinitis Pigmentosa: A Systematic Review and Meta-Analysis
Source: Front Med (Lausanne). 2022 May 16;9:895208. doi: 10.3389/fmed.2022.895208 (PMC9149278; doi:10.3389/fmed.2022.895208)
Supplement: Supplementary file 2 [file Data_Sheet_2.pdf]

Supplemental material for the paper "Management of cystoid macular edema in retinitis pigmentosa: a systematic review and meta-analysis"

Chen Chen, Xia Liu, and Xiaoyan Peng
*Correspondence:
Professor Xiaoyan Peng, MD, PhD E-mail: 74000041@ccmu.edu.cn

Search strategy for PubMed:

(retinitis pigmentosa OR rod-cone dystrophy OR rod-cone degeneration OR Tapetoretinal Degeneration OR Tapetoretinal Degenerations OR Pigmentary Retinopathy OR Pigmentary Retinopathies OR inherited retinal degenerations) AND (Macular Edema OR Macula edema OR Macular oedema OR macula oedema OR Cystoid Macular Edema OR Cystoid Macular oedema OR Cystoid Macular Dystrophy OR Central Retinal Edema)

Search strategy for Embase:

#1. ('retinitis'/exp OR retinitis) AND pigmentosa
#2. 'rod cone' AND dystrophy
#3. 'rod cone' AND degeneration
#4. tapetoretinal AND degeneration
#5. tapetoretinal AND degenerations
#6. pigmentary AND retinopathy
#7. pigmentary AND retinopathies
#8. retinopathies, AND pigmentary
#9. retinopathy, AND pigmentary
#10. inherited AND retinal AND degenerations
#11. #1 OR #2 OR #3 OR #4 OR #5 OR #6 OR #7 OR #8 OR #9 OR #10
#12. macular AND edema
#13. macula AND edema
#14. macular AND oedema
#15. macula AND oedema
#16. cystoid AND macular AND edema
#17. cystoid AND macular AND oedema
#18. cystoid AND macular AND dystrophy
#19. central AND retinal AND edema
#20. #12 OR #13 OR #14 OR #15 OR #16 OR #17 OR #18 OR #19
#21. #11 AND #20
#22. animal OR 'animal experiment'
#23. 'human'
#24. #22 AND #23
#25. #22 NOT #24
#26. #21 NOT #25

Search strategy for the Cochrane library:

#1 retinitis pigmentosa
#2 rod-cone dystrophy
#3 rod-cone degeneration
#4 Tapetoretinal Degeneration
#5 Tapetoretinal Degenerations
#6 Pigmentary Retinopathy
#7 Pigmentary Retinopathies
#8 Retinopathies, Pigmentary
#9 Retinopathy, Pigmentary
#10 inherited retinal degenerations
#11 #1 OR #2 OR #3 OR #4 OR #5 OR #6 OR #7 OR #8 OR #9 OR #10
#12 Macular Edema
#13 Macula edema
#14 Macular oedema
#15 macula oedema
#16 Cystoid Macular Edema
#17 Cystoid Macular oedema
#18 Cystoid Macular Dystrophy
#19 Central Retinal Edema
#20 #12 OR #13 OR #14 OR #15 OR #16 OR #17 OR #18 OR #19
#21 #11 AND #20

Search strategy for clinical trials.gov

In the box of "cindition or disease":
(retinitis pigmentosa OR rod-cone dystrophy OR rod-cone degeneration OR Tapetoretinal Degeneration OR Tapetoretinal Degenerations OR Pigmentary Retinopathy OR Pigmentary Retinopathies OR inherited retinal degenerations)
In the box of "other terms":
(Macular Edema OR Macula edema OR Macular oedema OR macula oedema OR Cystoid Macular Edema OR Cystoid Macular oedema OR Cystoid Macular Dystrophy OR Central Retinal Edema)

Search strategy for WHO ICTRP:

retinitis pigmentosa and macular edema
